# Multisite infection with Austrian syndrome caused by non-vaccine pneumococcal serotype—case report

**DOI:** 10.1128/asmcr.00061-25

**Published:** 2025-08-21

**Authors:** Frederik Juel Pontoppidan Børrild, Emil Fosbøl, Casper Roed, Melissa Hornbæk Øvre, Helle Brander Eriksen, Christian Kraef, Jannik Helweg-Larsen, Zitta Barrella Harboe, Christina Ekenberg

**Affiliations:** 1 Department of Pulmonary and Infectious Diseases, Copenhagen University Hospital, North Zealand, Denmark; 2 Department of Cardiology, Rigshospitalet, Copenhagen University Hospital 53167, Copenhagen, Denmark; 3 Department of Clinical Medicine, University of Copenhagen 4321, Copenhagen, Denmark; 4 Department of Clinical Microbiology, Copenhagen University Hospital 53167, Herlev-Gentofte, Herlev, Denmark; 5 Department of Infectious Diseases, Rigshospitalet, Copenhagen University Hospital 53167, Copenhagen, Denmark; 6 Department of Bacteria, Parasites and Fungi, Statens Serum Institut 4326, Copenhagen, Denmark; Vanderbilt University Medical Center, Nashville, Tennessee, USA

**Keywords:** invasive pneumococcal disease, pneumococcal infection, meningitis, endocarditis, serotype, *Streptococcus pneumoniae*, Austrian syndrome

## Abstract

**Background:**

Although rare, Austrian syndrome remains a life-threatening triad consisting of pneumonia, endocarditis, and meningitis caused by *Streptococcus pneumoniae*.

**Case Summary:**

We present a case of Austrian syndrome, further complicated by spondylodiscitis and endophthalmitis, in an immunocompromised male receiving methotrexate treatment for rheumatoid arthritis. The infection was caused by a non-vaccine pneumococcal serotype (23B). Susceptibility testing showed reduced susceptibility towards benzylpenicillin, an uncommon finding in Denmark.

**Conclusion:**

This case illustrates some of the challenges faced in managing invasive pneumococcal disease and underlines the importance of continued awareness of this disease, particularly in immunocompromised patients.

## INTRODUCTION

Due to the widespread use of β-lactam antibiotics and the introduction of pneumococcal vaccines, the incidence of invasive pneumococcal disease (IPD) has declined (1). Still, it remains associated with high morbidity and mortality, including in immunocompromised individuals (2). Austrian syndrome is a triad of meningitis, endocarditis, and pneumonia caused by *Streptococcus pneumoniae*. It is associated with a poor prognosis, and the severity of Austrian syndrome highlights the importance of early diagnosis and treatment in addition to timely vaccination. Furthermore, with the increased usage of pneumococcal vaccines, non-vaccine serotypes are emerging, emphasizing the need for continued surveillance. Here, we present a case of Austrian syndrome in a 65-year-old male undergoing treatment with methotrexate due to rheumatoid arthritis (RA).

## CASE PRESENTATION

**Fig 1:**
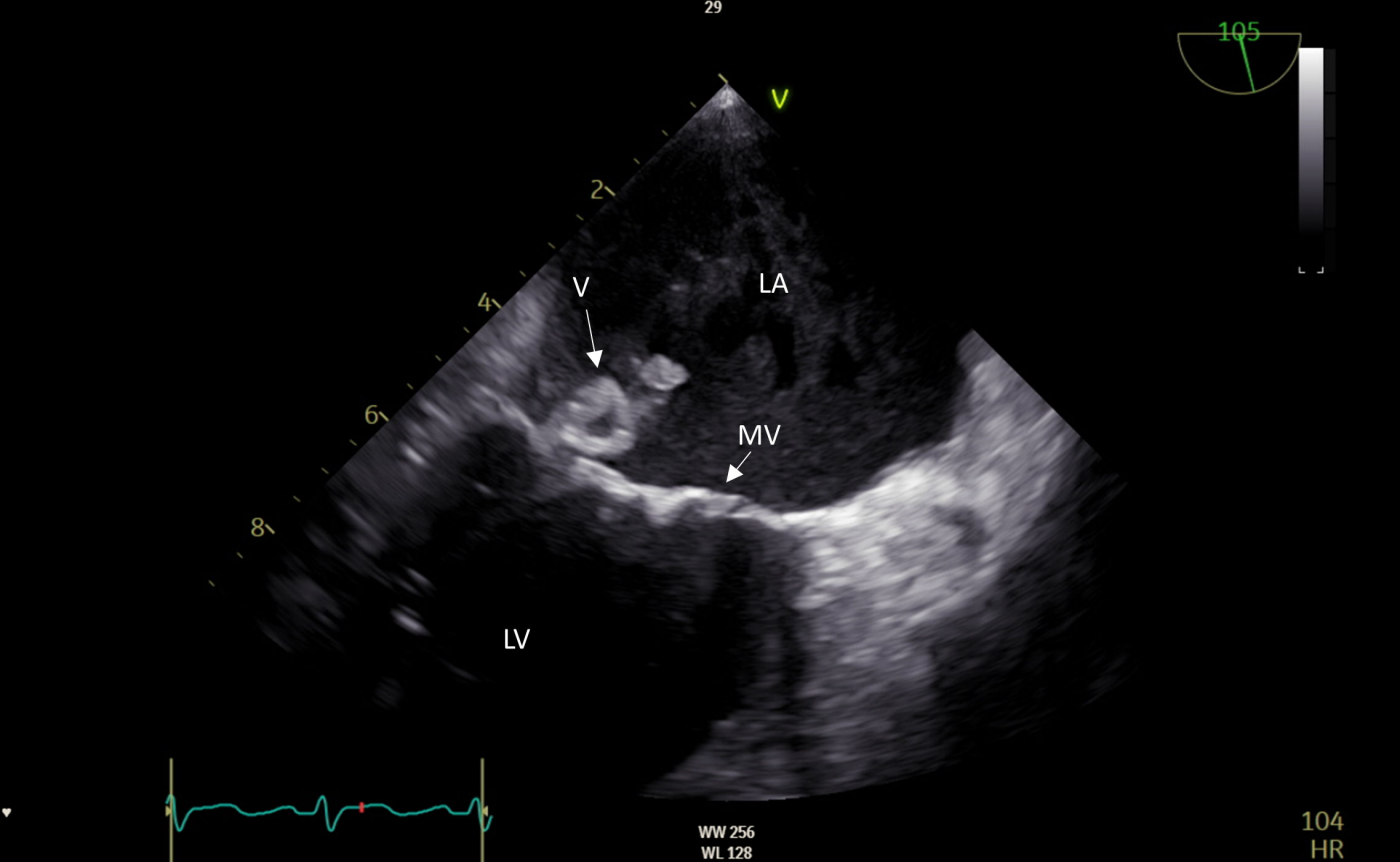
First transesophageal echocardiogram (TEE) of the patient. The TEE revealed a large vegetation of the posterior mitral leaflet. Further, in relation to the vegetation, the mitral valve was perforated, and the infection extended to the annulus of the valve, and a ruptured abscess in relation to the mitral valve was found. LA: left atrium, LV: left ventricle, MV: mitral valve, V: vegetation.

A 65-year-old male with a medical history of hypertension and rheumatoid arthritis (RA) was admitted to the hospital with a 14-day history of cough, malaise, and new-onset altered mental state. The patient received treatment with methotrexate 20 mg weekly, which is considered low-to-moderate dose (>20 mg weekly is considered moderate-severe immunosuppression) (3), and had 1 week prior to admission received a steroid injection, which may have caused a synergistic immunosuppressive effect. On examination, he appeared mildly disoriented, and a systolic murmur was noted upon auscultation. His vital signs were blood pressure of 113/75 mm Hg, pulse rate of 110, body temperature of 37.5°C, respiratory rate of 18, and oxygen saturation of 99% without supplementing oxygen. Initial blood tests showed a leukocyte count of 11.1 × 10^9^/L (3.5–8.8 × 10^9^/L), CRP of 341 mg/L (<10 mg/L), and elevated lactate dehydrogenase and liver enzymes. Given the concurrent confusion and subfebrile temperature, a lumbar puncture was done, revealing the cerebrospinal fluid (CSF) with neutrophilic pleocytosis with a white cell count of 2,240 cells/µL (<5 cells/µL), low glucose of 0.8 mmol/L (2.2–3.9 mmol/L), elevated protein of 3.1 g/L (0.15–0.5 g/L), and lactate of 12.4 mmol/L (1.1–2.4 mmol/L). Empiric treatment for bacterial meningitis was initiated, consisting of intravenously (IV) administered benzylpenicillin 1.8 g every 4 h, ceftriaxone 4 g once daily, and dexamethasone 10 mg every 6 h. Multiplex nucleic acid test (The BIOFIRE FILMARRAY Meningitis-Encephalitis Panel, BioMerieux) performed on CSF tested positive for *S. pneumoniae* within 2 h after the lumbar puncture. A computed tomography (CT) followed by magnetic resonance imaging (MRI) of the brain demonstrated small, scattered, primarily cortical infarctions in both hemispheres, suggesting septic emboli. The transthoracic echocardiogram (TTE) was inconclusive, while a transesophageal echocardiogram (TEE) revealed a large vegetation of the posterior mitral leaflet (Fig. 1). In relation to the vegetation, there was a perforation of the valve, and the infection extended to the annulus of the valve. Cardiac CT angiography (CTA) indicated a ruptured abscess in relation to the mitral valve, which the TEE confirmed. Furthermore, vegetations were also found on the tricuspid and on the aortic valves without substantial valve destruction. The patient underwent reconstructive surgery on both the mitral and the tricuspid valve. A follow-up TEE subsequently showed a minor residual perforation of the mitral valve, along with slight mitral regurgitation. Blood cultures yielded growth of *S. pneumoniae*, serotype 23B. Species identification was performed combining colony morphology, optochin susceptibility, and identification with MALDI-TOF MS Biotyper (Bruker, Bremen, Germany). A Gram stain was performed on CSF with no visible microorganisms. Several polynuclear lymphocytes were seen with a few mononuclear lymphocytes. *S. pneumoniae* was identified in CSF using the multiplex nucleic acid test; culture was negative. From mitral and tricuspidal tissues, *S. pneumoniae* was identified with an in-house 16S rRNA sequencing. No growth emerged.

Susceptibility testing was performed according to the EUCAST methodology for disc diffusion and gradient tests and interpreted using EUCAST breakpoint table 14.0 (https://www.eucast.org). For *S. pneumoniae,* screening with the oxacillin 1 µg disk diffusion is standard. For susceptible isolates (≥20 mm), benzylpenicillin, ceftriaxone, meropenem, and other β-lactam antibiotics can be reported susceptible. The blood culture isolate had decreased susceptibility to oxacillin (10 mm), so a supplementary gradient test was performed, which is recommended for meningitis isolates. With a minimum inhibitory concentration (MIC) of 0.250 mg/L towards benzylpenicillin, this is reported resistant according to meningitis breakpoints (S ≤ 0.06 mg/L). Ceftriaxone was susceptible with a MIC of 0.125 mg/L (S ≤ 0.5 mg/L). Supplementary susceptibility testing of the isolate was performed because of endocarditis (Table 1). Consequently, benzylpenicillin was discontinued, and moxifloxacin 400 mg twice daily per os (p.o.) was prescribed, in addition to ceftriaxone. At day 4 of hospitalization, the patient presented with visual hallucinations and blurry vision. Upon ophthalmologic examinations, the visual impairments were found to be caused by bilateral endophthalmitis. A positron emission tomography (PET) scan was performed to investigate additional infectious foci. Aside from a pneumonic infiltrate, it showed inflammatory alterations suggestive of spondylodiscitis at the Th12–L3 level.

**TABLE 1:** Antibiogram of the *Streptococcus pneumoniae* found in the blood cultures^*a*^

Antibiotic	Susceptibility	MIC
Penicillin	R	0.250 mg/L
Ceftriaxone	S	0.125 mg/L
Cefotaxime	S	0.125 mg/L
Moxifloxacin	S	0.250 mg/L
Linezolid	S	2.000 mg/L
Rifampicin	S	0.064 mg/L
Amoxicillin, p.o.^*b*^	N/A^*c*^	N/A

^*a*^ Penicillin, ceftriaxone, and cefotaxime were interpreted with meningitis breakpoint. Moxifloxacin, rifampicin, and linezolid were reported because of endocarditis. Susceptibility testing was interpreted using EUCAST breakpoint table 14.0. S: susceptible at standard doses, I: susceptible at increased doses, R: resistant, MIC: minimum inhibitory concentration.

^*b*^ Amoxicillin p.o. was not reported, but can be reported susceptible for non-meningitis isolates with an oxacillin zone ≥9 mm.

^*c*^ N/A: not applicable.

After 6 weeks of antibiotic treatment with ceftriaxone and moxifloxacin, the patient was discharged with amoxicillin 750 mg p.o. four times daily for six additional weeks to complete treatment for spondylodiscitis.

During follow-up consultations in the outpatient clinic, the patient kept gradually improving, and he resumed working. His visual complaints, however, are expected to be permanent to some extent. The patient has later been diagnosed with epilepsy, which has been attributed to the central nervous system involvement.

## DISCUSSION

We have presented a rare case of Austrian syndrome, a triad consisting of pneumonia, endocarditis, and meningitis caused by *S. pneumoniae*, further complicated by spondylodiscitis and endophthalmitis in an immunocompromised adult. Austrian syndrome, or Osler’s triad, is a rare phenomenon, as only 1%–3% of cases with bacterial endocarditis are caused by pneumococci (4), of whom less than 1% end up developing Austrian syndrome (5). The triad is associated with a poor prognosis, as pneumococcal endocarditis alone has a mortality rate ranging from 28% to 60% (4), while the fatality rates of pneumococcal meningitis have been reported to be 19% to 37% (6). Madu et al. conducted a systematic review of 71 cases of Austrian syndrome, reporting a case fatality of 28% in cases documented between 1991 and 2022, with the majority of studies (70%) published after 2010 (7).

The finding of *S. pneumoniae* with decreased susceptibility towards penicillin is a relatively uncommon finding in Denmark, as 94% of *S. pneumoniae* isolates in invasive infections (bacteremia and meningitis) are susceptible to benzylpenicillin (2023) (8). The pneumococcal isolate was identified as serotype 23B. In 2024 (data available up until October), six cases of IPD due to serotype 23B were registered in Denmark, of which only two were susceptible to penicillin (Statens Serum Institut, unpublished data).

Importantly, this serotype is not covered by any of the licensed pneumococcal vaccines (9). While the widespread availability of pneumococcal vaccines has proved to decrease the incidence of IPD caused by vaccine serotypes markedly, non-vaccine serotypes can cause severe disease (10, 11). In Denmark, the implementation of PCV7 and PCV13 in the Danish childhood vaccination program has decreased the incidence of vaccine serotypes significantly, although non-vaccine serotypes have increased (12). This emphasizes the importance of continuously surveying the distribution of pneumococcal serotypes causing IPD to ensure that future vaccines can be adjusted to cover the most prevalent serotypes. A Swedish study found that patients ≥65 years and patients with predisposing factors, such as immunosuppressive therapy, were more likely to have IPD caused by a non-vaccine serotype (13). Furthermore, serotype 23B has been found as a cause of severe IPD in previously healthy young, vaccinated adults (14). To our knowledge, it is not known if this specific serotype, or any other serotype in particular, is associated with the development of Austrian syndrome.

Of note, the patient in our case report had not previously received any pneumococcal vaccine but was vaccinated in the outpatient clinic after he had recovered. Unfortunately, several studies have shown that pneumococcal vaccine uptake in at-risk populations remains suboptimal (15) despite well-established guidelines (16). In Denmark, pneumococcal vaccination is recommended to high-risk individuals, including patients with rheumatic diseases receiving immunosuppressive therapy, such as methotrexate, preferably prior to initiation of immunosuppression. The cost is partially covered by a subsidy.

This case demonstrates the ongoing challenges of managing IPD. Regardless of vaccination status, awareness and suspicion of IPD should be upheld, particularly in—but not restricted to—the immunocompromised patients, in whom an atypical presentation of symptoms is not uncommon.
